# Supplementary material for: Novel Driver Strength Index highlights important cancer genes in TCGA PanCanAtlas patients
Source: PeerJ. 2022 Aug 11;10:e13860. doi: 10.7717/peerj.13860 (PMC9375969; doi:10.7717/peerj.13860)
Supplement: Supplemental Information 2 [file peerj-10-13860-s002.zip › COHORTS/genes plots/age/2021_8_16_14_9_distribution_cohorts_85_.pdf]

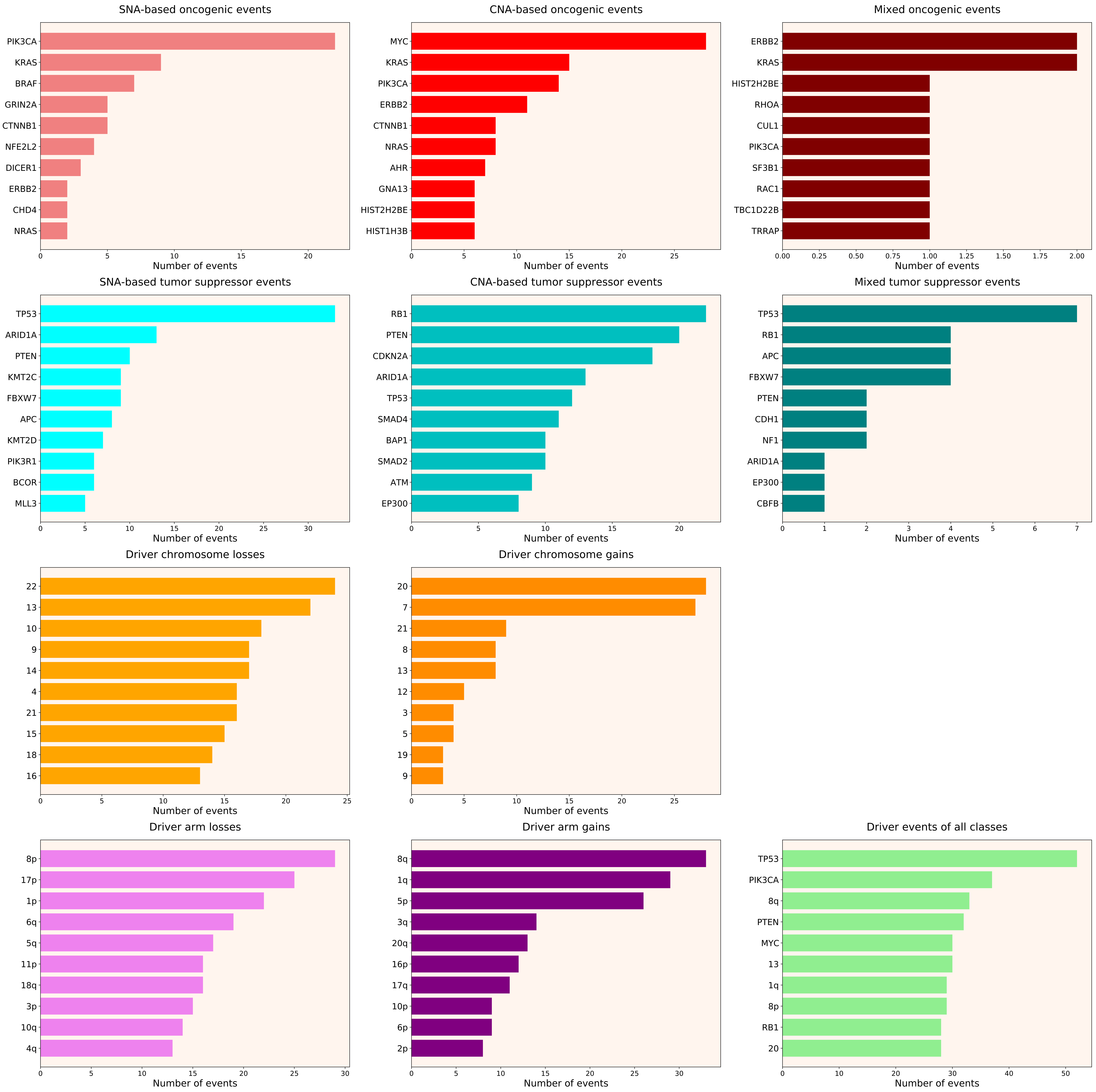
SNA-based oncogenic events
PIK3CA
KRAS
BRAF
GRIN2A
CTNNB1
NFE2L2
DICER1
ERBB2
CHD4
NRAS
0
5
10
15
20
Number of events
CNA-based oncogenic events
MYC
KRAS
PIK3CA
ERBB2
CTNNB1
NRAS
AHR
GNA13
HIST2H2BE
HIST1H3B
0
5
10
15
20
25
Number of events
Mixed oncogenic events
ERBB2
KRAS
HIST2H2BE
RHOA
CUL1
PIK3CA
SF3B1
RAC1
TBC1D22B
TRRAP
0.00
0.25
0.50
0.75
1.00
1.25
1.50
1.75
2.00
Number of events
SNA-based tumor suppressor events
TP53
ARID1A
PTEN
KMT2C
FBXW7
APC
KMT2D
PIK3R1
BCOR
MLL3
0
5
10
15
20
25
30
Number of events
CNA-based tumor suppressor events
RB1
PTEN
CDKN2A
ARID1A
TP53
SMAD4
BAP1
SMAD2
ATM
EP300
0
5
10
15
20
Number of events
Mixed tumor suppressor events
TP53
RB1
APC
FBXW7
PTEN
CDH1
NF1
ARID1A
EP300
CBFB
0
1
2
3
4
5
6
7
Number of events
Driver chromosome losses
22
13
10
9
14
4
21
15
18
16
0
5
10
15
20
25
Number of events
Driver chromosome gains
20
7
21
8
13
12
3
5
19
9
0
5
10
15
20
25
Number of events
Driver arm losses
8p
17p
1p
6q
5q
11p
18q
3p
10q
4q
0
5
10
15
20
25
30
Number of events
Driver arm gains
8q
1q
5p
3q
20q
16p
17q
10p
6p
2p
0
5
10
15
20
25
30
Number of events
Driver events of all classes
TP53
PIK3CA
8q
PTEN
MYC
13
1q
8p
RB1
20
0
10
20
30
40
50
Number of events
